# Multi-omics integration reveals inhibitory effects of carbon ion radiation on lung adenocarcinoma proliferation

**DOI:** 10.3389/fpubh.2026.1802182

**Published:** 2026-07-03

**Authors:** Zhen Yang, Shuangwu Feng, Xiucai Ma, Lina Wang, Shilong Sun, Zhiqiang Liu, Xuejiao Tian, Yichao Geng, Qiuning Zhang

**Affiliations:** 1 Gansu Provincial Peoples Hospital, Lanzhou, China; 2 Institute of Modern Physics, Chinese Academy of Sciences, Lanzhou, China; 3 School of Public Health, Gansu University of Chinese Medicine, Lanzhou, China; 4 The First School of Clinical Medicine, Lanzhou University, Lanzhou, China; 5 Department of Oncology, The Affiliated Hospital of Guizhou Medical University, Guiyang, China; 6 Lanzhou Ion Therapy Co., Ltd, Lanzhou, China

**Keywords:** carbon ions, GCH1, lung adenocarcinoma, multi-omics, proliferation inhibition

## Abstract

**Objective:**

To analyze the inhibitory effect of carbon ion (C-ion) radiation-induced biological effects on the proliferation of lung adenocarcinoma (LUAD) by multi-omics integration.

**Methods:**

We investigated C-ion radiation effects on LUAD cellular responses using A549 and LLC cell lines. Clonogenic survival assays, DNA damage, and metastasis quantified radiation sensitivity, while a multi-cell co-culture system (A549/Beas-2B/LLC/MLE-12) differentiated direct vs. bystander effects. Integrated transcriptomics and targeted metabolomics identified radiation-responsive genes/metabolites, with pathway analysis conducted through MetaboAnalyst 6.0. Clinical relevance of CPT1, GCH1, and EPAS1 was assessed using UCSC Xena and Kaplan-Meier Plotter survival data. Radiation-induced molecular changes were validated by RT-qPCR and immunoblotting across cell types. An A549 tumor-bearing mouse model was established, and the growth and tumor size of the tumor-bearing mice were observed after irradiation with C-ions. Blood was taken from mice after anesthesia and necropsy to detect changes in the major differential metabolism factor arachidonic acid (AA), and tumor tissues were examined to detect changes in the expression of CPT1, GCH1, and EPAS1 in the tissues.

**Results:**

C-ion irradiation exerts a dual anti-proliferative effect on lung adenocarcinoma cells: it directly induces DNA damage (increased γ-H2AX/53BP1 foci), suppresses clonogenic survival, and inhibits tumor cell migration and invasion (*p* < 0.05); meanwhile, it amplifies the bystander effect from co-cultured normal cells through metabolic reprogramming, thereby enhancing overall cytotoxicity. Transcriptomic-metabolomic integration identified CPT1, GCH1, and EPAS1 as central regulators of radiation-induced metabolic suppression, with AA, 3-hydroxytetradecanoic acid, and 2-methylglutaric acid constituting critical downstream mediators. Database analysis revealed that the differential expression of CPT1, GCH1, and EPAS1 was correlated with the prognostic survival status of patients with lung adenocarcinoma. Carbon ion irradiation downregulated the expression of GCH1 and upregulated CPT1 and EPAS1. Animal experiments demonstrated that carbon ions markedly inhibited tumor proliferation in tumor-bearing mice. The level of arachidonic acid in mouse blood was significantly increased (*p* < 0.05). Carbon ion radiation suppressed GCH1 expression and promoted the expression of CPT1 and EPAS1 in tumor tissues (*p* < 0.05).

**Conclusion:**

C-ion irradiation suppresses lung adenocarcinoma through a dual mechanism involving direct induction of cellular DNA damage and a metabolically enhanced bystander effect, driven by the CPT1/GCH1/EPAS1 regulatory axis, with arachidonic acid serving as a key downstream mediator.

## 1 Introduction

C-ion therapy, leveraging its dual advantages of physical precision (dose-depth distribution) and enhanced biological efficacy, effectively spares organs at risk and shows significant clinical benefits in locally advanced or radioresistant non-small cell lung cancer (NSCLC), though its underlying mechanisms remain elusive. Lung cancer is one of the most common malignant tumors globally. NSCLC is the main pathological type of lung cancer, accounting for about 80%−85% of all cases of lung cancer (1). High linear energy transfer (LET) radiation demonstrates superior local control rates, reduced radiation exposure to adjacent organs at risk, and lower adverse event rates in the clinical management of NSCLC. Clinical trials in early-stage lung cancer have demonstrated excellent local control even with single-fraction carbon ion radiotherapy (CIRT) (2) or when CIRT is used for in-field recurrence after prior radiotherapy in NSCLC (3). In a study of 64 patients with locally advanced NSCLC treated with CIRT, the 2-year overall survival (OS) rate was 62.2%, the 2-year local control rate was 81.8%, and the 2-year progression-free survival (PFS) rate was 42.3% (4). In NSCLC patients with pulmonary oligometastases, CIRT achieved a local control rate of 92.4% with mild adverse effects (5). It directly targets tumor tissue to disrupt DNA integrity and function, thereby suppressing tumor cell division and proliferation. It can also induce indirect effects (e.g., reactive oxygen species such as hydroxyl radicals and hydrogen peroxide), damaging nucleic acids, proteins, and lipids to cause cellular injury or death (6). To date, most radiobiological investigations have relied on single-omic profiling strategies, which are insufficient to fully dissect the complex and heterogeneous nature of radiation-induced biological responses. Compared with single-omic analysis, multi-omic integration can yield comprehensive biological information and uncover novel mechanistic insights that are inaccessible via individual single-omic approaches (7). Nevertheless, current studies of radiation response remain predominantly confined to single-omic analyses, which fail to delineate the synergistic crosstalk between gene regulatory networks and dynamic metabolic pathways. Here, we utilized transcriptomics and metabolomics to present a multi-omics approach for better understanding the biological effects induced by C-ion radiation. In matched pre- and post-radiotherapy patient samples, elevated levels of 4-Hydroxynonenal (4-HNE) a lipid peroxidation byproduct-were observed post-treatment, with its increase positively correlated with therapeutic efficacy and disease-free survival (DFS) (8). Alterations in the lipid metabolism of tumor cells have gradually become of concern due to the fact that lipid metabolism is closely associated with tumor development (9). Our study demonstrates that C-ion radiation inhibits NSCLC proliferation by altering transcriptomic and metabolomic profiles (10, 11). However, the direct/indirect mechanisms underlying its tumor-suppressive effects remain incompletely characterized. These findings provide a theoretical foundation for advancing metabolism-targeted radio sensitization strategies and improving the efficacy of precision radiotherapy in NSCLC.

## 2 Methods

### 2.1 Cell culture and co-culture model

The American Type Culture Collection (ATCC) provided human LUAD cell line A549, Beas-2B, Lewis (LLC), and MLE-12 cells for this study. The cells (mycoplasma-free) were cultured in RPMI 1640 medium (Hyclone, USA). supplemented with 10% fetal bovine serum (Gibco, Invitrogen, Carlsbad, California, USA) and a penicillin-streptomycin solution (penicillin 100 U/mL, streptomycin 100 mg/mL) at 37 °C in a humidified atmosphere containing 5% CO_2_ and 95% air. The medium was changed daily, and all experiments were performed using cells in the logarithmic growth phase (× 107 cells).

Co-culture experiments were performed using a Transwell system with 0.4-μm pore inserts (Corning, NY, USA). A549 and LLC cells were seeded in the upper chamber, while BEAS-2B and MLE-12 cells were seeded in the lower chamber; both cell populations were cultured in RPMI-1640 medium. Following carbon ion irradiation (5.16 Gy), the BEAS-2B/MLE-12 cells in the lower chamber were co-cultured with the A549/LLC cells in the upper chamber for 48 h to evaluate the impact of metabolites released by the irradiated BEAS-2B and MLE-12 cells on A549 and LLC cells.

### 2.2 Irradiation conditions

Heavy ions were obtained by irradiating the cells with a C-ion (^12^C^6+^) beam from the Deep Therapeutic Terminal of the Institute of Modern Physics, Chinese Academy of Sciences (HIRFL-CSR). (Ray parameters: 100 MeV /u energy, 1 Gy/min dose rate, 5 mm wide Bragg peak, LET: 30 keV/um, 5 cm × 5 cm radiation field) to irradiate the cells. Building on our prior determination of the relative biological effectiveness dose for A549 cells, 5.16 Gy C-ion irradiation alters proliferation-associated gene expression (11, 12), whereas 2 Gy induces metabolomic shifts in normal lung epithelial cells (10). The irradiation doses were 0, 2, and 5.16 Gy.

### 2.3 Clonogenic assay and cell proliferation

Clonogenic survival was assessed by colony formation assay. Cells were seeded in six-well plates and, 24 h later, irradiated with single doses of 0 and 5.16 Gy. Cultures were then incubated for 10 days to allow colony growth. Colonies containing more than 50 cells were scored as survivors. Surviving fractions of irradiated cells were normalized to the plating efficiency of unirradiated controls.

EdU kit (RiboBio, China) was used for the detection of cell proliferation. Briefly, A549 and LLC lung adenocarcinoma cells were irradiated with carbon ions at doses of 0 and 5.16 Gy. After irradiation, the cells were harvested and seeded into 24-well plates at a density of 2 × 104 cells per well. Subsequently, 50 mM EdU solution was added to the culture medium. After 2 h of incubation, then the cells were fixed with 4% paraformaldehyde (PFA) and further stained with Apollo 567 Solution. After that, Hoechst 33342 (10 mg/ml) was selected to label nucleic acid. Images were obtained with an Olympus microscope (Olympus, Tokyo, Japan). The czaiell proliferation inhibition rate was assessed by detecting fluorescently labeled EdU-positive cells and calculating their proportion of the total cell count to evaluate cell proliferation levels.

### 2.4 Cell migration and invasion

The wound healing assay was performed to evaluate the migration ability of lung cancer cells (A549 and LLC). Cells were seeded in 6-well plates and cultured in RPMI-1640 medium supplemented with 10% fetal bovine serum until they reached approximately 90% confluence. Several straight scratches were created on the cell monolayer using a 10 μl pipette tip. The plates were then washed with PBS to remove non-adherent cells, and the medium was replaced with serum-free DMEM. After 48 h of incubation, images of the migrated cells were captured. The distance between the two cell-free edges was measured using ImageJ 1.53c software.

The 24-well Transwell chamber (Corning, NY, USA) inserts with 8-μm pore size polycarbonate membrane with Matrigel (BD Biosciences, San Jose, USA) was used to analyze the invasion of tumor cells. After 48 h of incubation, cells and Matrigel on the upper surface of the membrane were carefully removed using a cotton swab. The cells that had migrated or invaded to the lower side of the membrane were then fixed with 4% paraformaldehyde (Sigma-Aldrich, Missouri, USA), and stained with 0.5% crystal violet. Finally, stained cells in five randomly selected fields were counted and photographed under an inverted microscope at 200 × magnification for quantitative analysis.

### 2.5 Quantification of γH2AX and 53BP1 foci

Cells at 1 × 105 cells/mL (A549 and LLC) were irradiated with carbon-ion beams, then spun onto slides, fixed with 4% paraformaldehyde in PBS, permeabilized with 0.1% Triton X-100 in PBS, and blocked with Odyssey^®^ blocking buffer. γH2AX, and 53BP1 foci were detected using the following primary antibodies: rabbit monoclonal anti-γH2AX (ab26350, Abcam, USA) (1:200 dilution), and rabbit polyclonal anti-53BP1 (ab175933, Abcam, USA) (1:200 dilution). Goat Anti Rabbit IgG (H+L)-Alexa Fluor 594 (1:200) and Goat Anti Mouse IgG (H+L) (Proteintech, China)-Alexa Fluor 594 (Immunoway, California, USA) (1:200 dilution) served as secondary antibodies. Nuclei were counterstained with DAPI (blue). Cells containing more than four γH2AX, 53BP1 foci were considered positive. γH2AX and 53BP1 foci were quantified in dual-channel confocal images using ImageJ. A549 and LLC nuclei were identified by thresholding the DAPI signal. Analysis of γH2AX was performed using a Leica DM5000 B fluorescence microscope (Leica Microsystems, Germany). For each sample, 100 cells were evaluated, and the number of γH2AX foci was counted manually. Statistical analysis was based on the number of γH2AX foci per cell (γH2AX foci/cell) (13, 14).

### 2.6 RNA-seq data processing and analysis

Total RNA was isolated using Trizol reagent (Invitrogen, Carlsbad, California, USA) according to the manufacturer's instructions. For next-generation sequencing, RNA was extracted using the PicoPure RNA Isolation Kit (Thermo Fisher Scientific, Massachusetts, USA), following the manufacturer's protocol. cDNA was synthesized using mRNA as the template. Second-strand synthesis and mRNA removal generated double-stranded cDNA (dscDNA), which was then purified, end-repaired, ligated with adapters, and subjected to PCR enrichment. The library was amplified by PCR, purified, and subjected to quality control.

For transcriptomic data, quality control, genome mapping, and read counting were performed. RNA-seq reads were assessed for quality using FastQC software. Paired-end sequencing (2 × 150 bp) was carried out on the Illumina Novaseq™ 6000 platform. Differentially expressed genes were screened using a fold change threshold of ≥2 (|log2FC| ≥ 1) and a *q-value* < 0.05 (*q-value* representing the adjusted *p-value*). Each comparison group consisted of three biological replicates. Transcript quantification and differential expression analysis were conducted using RSEM and edgeR, respectively, within the Bioconductor package in R. Analyses included enrichment analysis of differentially expressed genes, Gene Set Enrichment Analysis (GSEA), and assessment of alternative splicing events at the transcriptome level. Batch effects across six RNA-seq batches were corrected using canonical correlation analysis to identify linear combinations of features exhibiting the highest correlation across datasets.

### 2.7 Analysis of lipid metabolism

Collection of culture medium after irradiation of Beas-2B cells. The collected samples were thawed on ice, and metabolite were extracted with lipid extraction buffer. All samples were acquired by the LC-MS system followed machine orders. Preparation of lipid standards Lipid internal standard, PE (17:1/12:0), was used for adjustment of possible inter- and intra-assay variances (internal standardization). Firstly, all chromatographic separations were performed using an ACQUITY UPLC System (Waters, Milford, USA). A Kinetex UPLC C18 column (100 mm × 2.1 mm, 100 A, phenomenex, UK) was used for the reversed phase separation. A high-resolution tandem mass spectrometer TripleTOF6600 (SCIEX, Framingham, USA) was used to detect metabolites eluted form the column. The Q-TOF was operated in both positive and negative ion modes. Each group consists of 6 replicates. Raw data were processed using LipidSearch software (version 4.1; Thermo Fisher Scientific, Massachusetts, USA) for peak extraction, alignment, identification (based on MS/MS spectra), and quantification, with parameters set as follows: precursor tolerance, 5 ppm; product tolerance, 5 ppm; and product ion threshold, 5%. Metabolite identification was further validated by accurate mass matching (error < 30 ppm) and MS/MS spectral matching against the Human Metabolome Database (HMDB; http://www.hmdb.ca), with a confidence threshold of ≥95% (*p* ≥ 0.05). To minimize signal intensity drift over time, QC-based robust LOESS signal correction was used to fit to the QC data. Finally, features with a CV of >30% within the pooled QC samples were removed. The metabolomics data were log2-transformed and normalized with internal standards on a per-sample, per-method basis. Univariate and multivariate statistical analyses were performed to identify differentially abundant lipid species. Univariate analysis included fold change (FC) analysis and Student's *t*-test. Multivariate analysis comprised unsupervised principal component analysis (PCA), partial least squares discriminant analysis (PLS-DA), and orthogonal partial least squares discriminant analysis. Differential lipid features were defined using the following criteria: *p-value* < 0.05, variable importance in projection (VIP) > 1.0, and fold change > 1.5 or < 0.67. The overlap of significantly altered lipid species across comparison groups was visualized using Venn diagrams.

### 2.8 Database were downloaded for gene expression analyses and survival analyses

The *expression* of *CPT1, GCH1*, and *EPAS1* in The Cancer Genome Atlas (TCGA) *database* was analyzed using *UALCAN database* (http://ualcan.path.uab.edu/analysis.html) (15). The Kaplan–Meier plotter (http://kmplot.com/analysis/) was used to assess the prognostic impact of *CPT1, GCH1*, and *EPAS1* expression on overall survival (OS) in lung cancer. The analysis included expression and clinical data from 2,166 patients, integrated from GEO and TCGA within the database. Patients were dichotomized into high- and low-expression groups based on the median expression of each gene. Kaplan–Meier survival curves were generated. Hazard ratio (HR) and its corresponding 95% CI were estimated through Cox proportional hazards model, and *p-value* was generated from the Log rank test. Subgroup analyses by disease stage were also performed (16).

### 2.9 Tumor xenograft was undertaken in nude mice

This study was performed in strict accordance with the recommendations of the National Institutes of Health's Guide for the Care and Use of Laboratory Animals. Male BALB/c nude mice (6-week-old) were maintained from Sibeifu Beijing Biotechnology Co. Ltd. (Beijing, China). It was randomly allocated into two experimental groups (*n* = 6/group) using computer-generated randomization. Subcutaneous injection of A549 cells (1 × 10^6^ in 0.1 ml PBS) was performed on nude mice. Tumor dimensions (minor axis *a* and major axis *b*) were measured thrice weekly using digital calipers, with volumes calculated as *V* = *(a* × *b*^2^*)/2* (mm3). Anesthetized transplanted mice were locally irradiated with 5.16 Gy of C-ion. Twenty-one days post injection, the mice were sacrificed to determine the tumor volumes and were photographed. Tumor-transplanted mice were ethically sacrificed when the tumor volume reached at 2,000 mm^3^ or a tumor burden greater than 10% of the body weight.

### 2.10 Enzyme-linked immunosorbent assay (ELISA)

The procedure was with reference to the instructions of the kit. Mouse AA ELISA kits were purchased from Solarbio Biotechnology Co., Ltd. (China). The sensitivity of the assay was 1 pg/ml, according to the manufacturer's instructions.

### 2.11 RNA extraction and quantitative PCR

Total RNA was extracted from the cells using TRIzol reagent and RNA concentration was measured using a Qubit 8000 (Thermo Fisher Scientific, Massachusetts, USA). For RNA extraction, to each culture dish, 1 ml of TRIzol reagent (Invitrogen, Carlsbad, California, USA) was added and the cells were scraped with a cell scraper. The cells were collected in a 1.5 ml EP tube and placed on ice for 10 min. RNA was extracted using chloroform, followed by extraction and precipitation of the aqueous phase with isopropanol. The supernatant was discarded, and the precipitate was washed twice with 80% ethanol, then dissolved in 10 μl of RNAse-free water. After extraction, RNA quantity, purity, and quality were determined by NanoDrop 1000 spectrophotometer (Thermo Fisher Scientific, Massachusetts, USA). Complementary DNA (cDNA) was synthesized using iScript Reverse Transcriptase kit with 1 μg of purified RNA. Quantitative real-time PCR (qPCR) was performed in triplicate^*^ using a QuantStudio™ 6 Pro System (Applied Biosystems, Waltham, MA, USA) with Hieff^®^ qPCR SYBR Green Master Mix. Cycling parameters were as follows: initial denaturation at 95 °C for 60 s, 95 °C for 10 s, 58 °C for 20 s, and 72 °C for 20 s. Forty cycles were performed. GAPDH was selected as the endogenous control based on stability validation across experimental conditions. Relative gene expression was calculated using the comparative 2^(−Δ*ΔCt*)^ method^*^: ΔCt = Ct (target) – Ct (GAPDH), ΔΔCt = ΔCt (treatment) – ΔCt (Control).

### 2.12 Western blotting analysis

Total protein was extracted using ice-cold RIPA lysis buffer (Thermo Fisher Scientific, Massachusetts, USA) supplemented with 1 × Halt™ Protease/Phosphatase Inhibitor Cocktail. Following homogenization, centrifugation (3,000 × g, 4 °C, 20 min) was performed and the supernatant was collected. Protein concentration was determined via BCA assay with bovine serum albumin (BSA) as standard. Equal amounts of protein lysates were resolved on 10% gradient SDS-PAGE gels under reducing conditions and transferred onto 0.45 μm PVDF membranes (Millipore, Bedford, MA, USA) using a Trans-Blot^®^ Turbo™ system. Antibodies were used for immunoblotting: polyclonal CPT1A (88 kDa, 1:1,000), GCH1 (28 kDa, 1:3,000), and EPAS1 (96 kDa, 1:1,500) antibodies were purchased from Proteintech (Cat# 15184-1-AP, Cat# 28501-1-AP, and Cat# 26422-1-AP). And antibody and horseradish peroxidase (HRP)-conjugated secondary antibody (anti-rabbit antibody) were purchased from Proteintech (Cat.No.66009-1-AP). The band intensities were quantified using ImageJ 1.53c software. Protein expression was quantified and standardized to the expression of actin protein. The values of the relative expression were obtained against the control treatments.

### 2.13 Immunohistochemical staining (IHC)

Tumor tissues from A549 tumor-bearing mice were fixed in 4% paraformaldehyde and embedded in paraffin. Sections were cut at 4 μm thickness and subjected to immunohistochemical staining for CPT1A (Proteintech, 1:1,000), GCH1 (Proteintech, 1:400), and EPAS1 (Proteintech, 1:500), with DAB used for chromogenic detection. Immunohistochemical staining was performed using a Leica automatic immunohistochemical staining machine. Protein expression levels were quantified based on the immunoreactivity score.

### 2.14 Statistical analysis

The experiments were conducted in triplicate unless otherwise specified. Statistical analysis was carried out using Prism statistical analysis software. Data without indications were analyzed by one-way ANOVA, Tukey *post hoc* test. A value of *p* < 0.05 was regarded as statistically significant. Animals were randomly allocated in each group using randomizer. Each treatment had three biological replicates, along with three technical replicates/biological replicates. Results were expressed as mean ± SD.

## 3 Results

### 3.1 C-ion radiation inhibits LUAD cell proliferation

**Figure 1:**
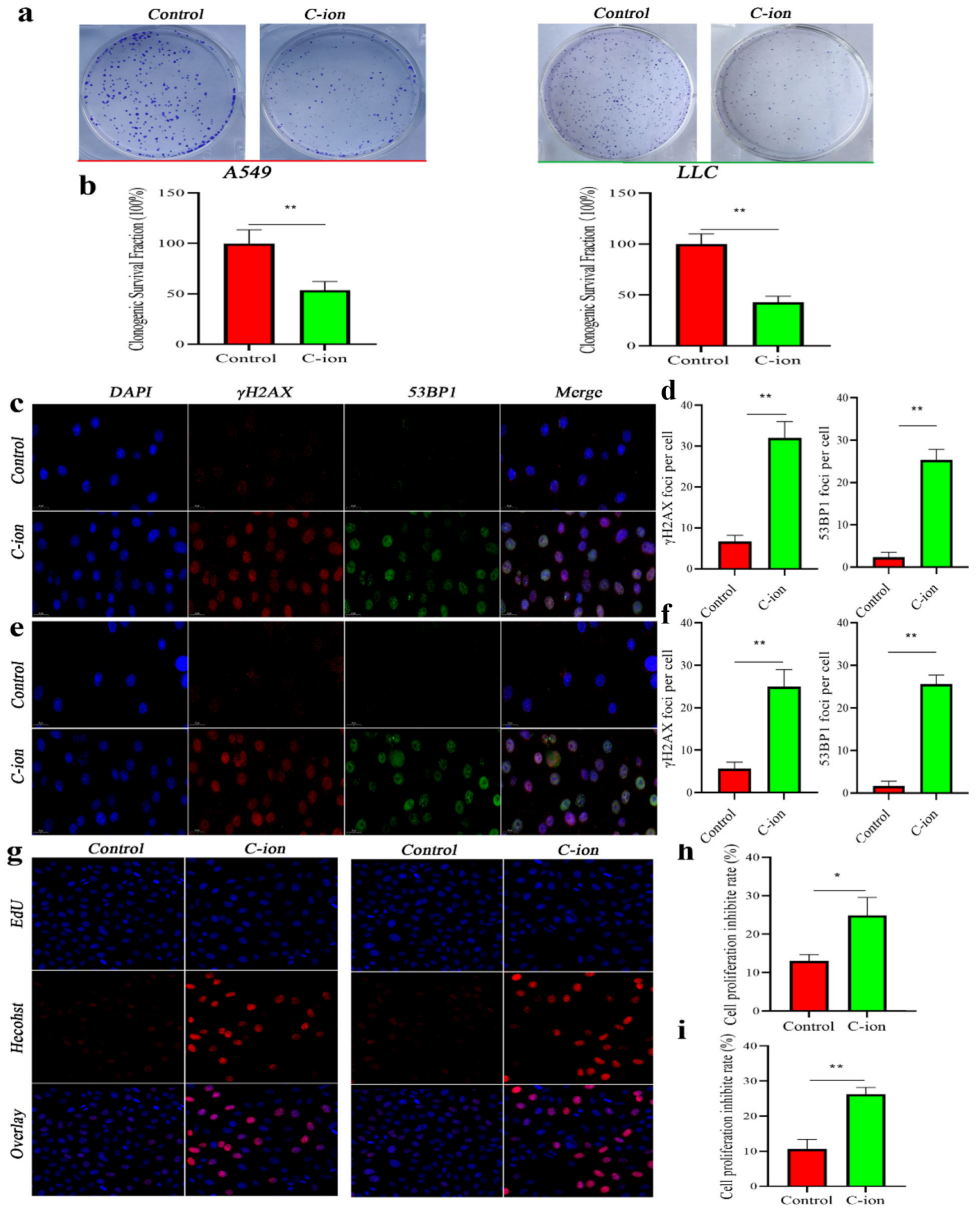
C-ion irradiation (5.16 Gy) suppresses lung adenocarcinoma cell proliferation. **(a)** Clonogenic formation in irradiated A549 and LLC cells. Number of biological replicates (*n*) = 3. **(b)** Clonogenic survival fractions of A549 and LLC cells post-C-ion irradiation. **(c**, **d)** After irradiation (5.16 Gy), A549 cells were staining and analysis for γ-H2AX and 53BP1 (*n* = 3). **(e**, **f)** After irradiation (5.16 Gy), LLC cells were staining and analysis for γ-H2AX and 53BP1. **(g)** EdU proliferative staining of irradiated A549 and LLC cells (*n* = 3). **(h**, **i)** Proliferative inhibition rates in irradiated A549 **(h)** and LLC **(i)** cells. *p < 0.05, **p < 0.01 vs. control. Bars represent mean ± SD.

For clonogenic survival assay, seeded 1 × 10^3^cells in each well of 6-well plate. After 12 days, fixed cell colonies in 10% formalin and stained with crystal violet (0.1% w/v). A549 and LLC lung adenocarcinoma cells were irradiated with 5.16 Gy C-ions to assess clonogenic survival. C-ion irradiation significantly reduced colony formation in both cell lines compared to controls (Figure 1a), with marked decreases in clonogenic survival fractions (*p* < 0.05, Figure 1b). Furthermore, we examined the expression levels of DNA damage markers (γ-H2AX and 53BP1) in tumor cells (A549) at 2 h post-irradiation (Figure 1c). 53BP1/γ-H2AX Foci Irradiation of A549 cells with 5.16 Gy resulted in a statistically significant increase in 53BP1/γ-H2AX foci dealing with radiation-induced DSBs (*p* < 0.05, Figure 1d). These results were also confirmed in LLC cells (Figure 1e). A significant increase in the number of 53BP1/γ-H2AX foci was observed following carbon ion irradiation (*p* < 0.05, Figure 1f). EdU staining at 48 h post-irradiation revealed diminished proliferative capacity (Figure 1g). Further analysis demonstrated that C-ions robustly inhibited proliferative activity in A549 and LLC cells (*p* < 0.05, Figures 1h, i), confirming their potent anti-proliferative effects in LUAD.

### 3.2 Effect of co-culture on the proliferation of LUAD cells

**Figure 2:**
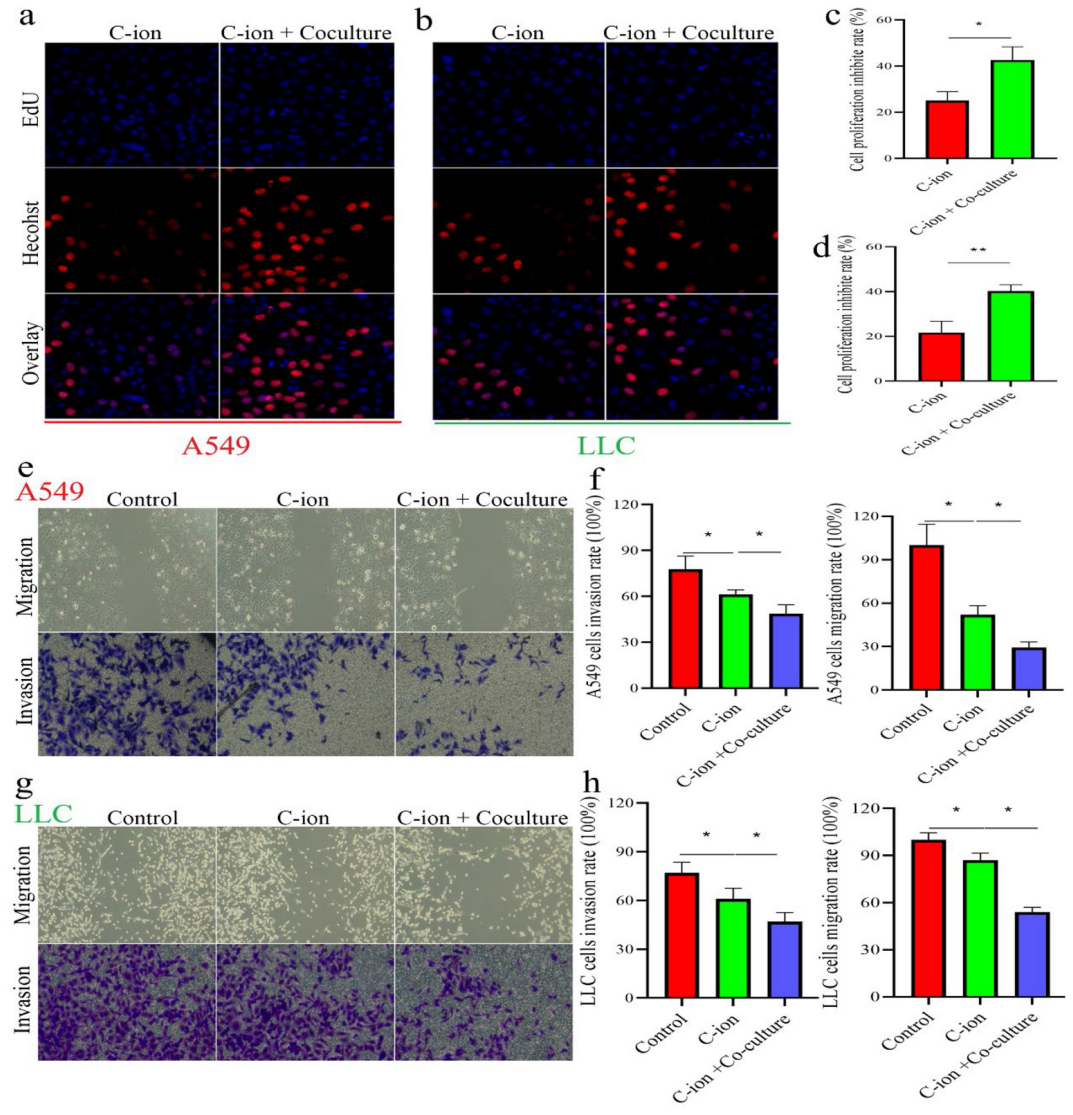
Inhibitory effects of C-ion irradiation on lung adenocarcinoma cell proliferation in co-culture systems (48 h). **(a**, **b)** EdU staining of A549 and LLC cells post-co-culture (*n* = 3). **(c**, **d)** Analysis of C-ion-induced proliferative inhibition rates in A549 and LLC cells within co-culture systems. **(e)** C-ion combined with Co-culture inhibits A549 cell migration, invasion (*n* = 3). **(f)** A549 cell migration and invasion analysis. **(g)** C-ion combined with Co-culture inhibits LLC cell migration, invasion (*n* = 3). **(h)** LLC cell migration and invasion analysis.). Bars represent mean ± SD. *p < 0.05, **p < 0.01 vs. control.

A Transwell co-culture system was established with LUAD cells (A549 and LLC) in the lower chamber and normal lung cells (Beas-2B and MLE-12) in the upper chamber. Tumor and normal cells were irradiated with 5.16 or 2 Gy C-ions, respectively. After 48 h of co-culture, tumor cell proliferative activity was assessed. Proliferative staining of lung adenocarcinoma cells post-irradiation and co-culture revealed suppressed growth in A549 and LLC cells (Figures 2a, b). Staining intensity analysis demonstrated that C-ion irradiation combined with co-culture significantly reduced A549 cell proliferative activity compared to irradiation alone (*p* < 0.05, Figure 2c), the results showed the same tendency in LLC cells (*p* < 0.01, Figure 2d). We further investigated the effects of carbon ion irradiation and its combination with the co-culture system on the metastatic capacity of tumor cells. The results from scratch wound healing and Transwell assays demonstrated that carbon ion irradiation alone and in combination with co-culture inhibited the migration and invasion of A549 cells (Figure 2e). Compared with the control group, the carbon ion treatment group significantly suppressed the migration and invasion rates of A549 cells (*p* < 0.05, Figure 2f). Similarly, consistent results were observed in LLC cells (Figure 2g), indicating that carbon ion irradiation combined with co-culture can significantly inhibit the metastasis of lung cancer cells (*p* < 0.05, Figure 2h). Alternatively, we further discovered that LLC cells exhibit stronger migration ability than A549 cells *in vitro*, but their invasion abilities are comparable.

### 3.3 C-ion irradiation induces transcriptomic changes in lung adenocarcinoma

**Figure 3:**
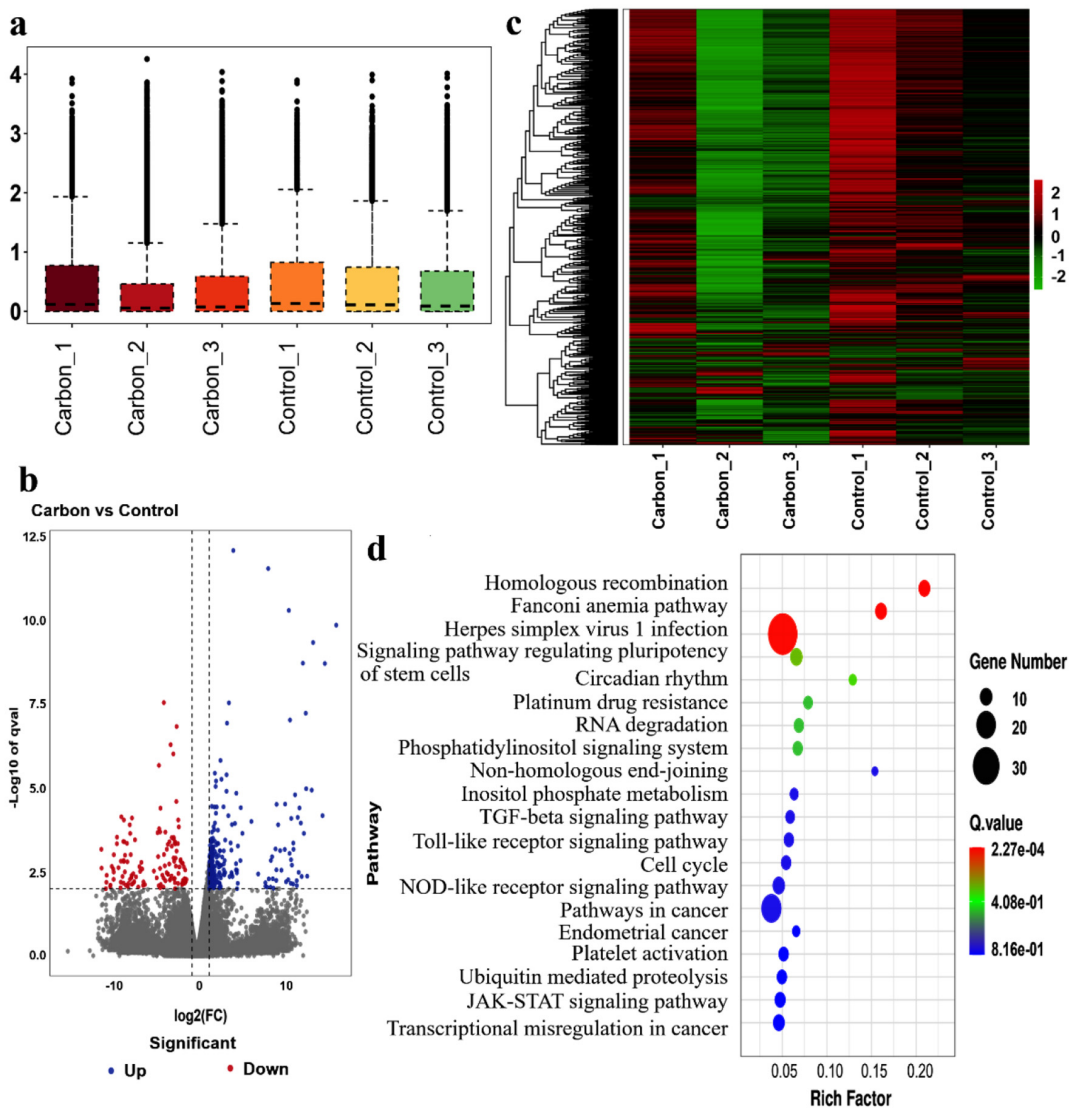
Transcriptomic analysis of A549 cells 48 h after C-ion irradiation (5.16 Gy). **(a)** Global distribution of intergroup gene expression (*n* = 3). **(b)** Differential gene analysis post-C-ion irradiation (*n* = 3). **(c)** Clustering analysis of differentially expressed genes (*n* = 3). **(d)** GO functional enrichment of irradiation-induced differentially expressed genes.

A549 cells irradiated with 5.16 Gy C-ions underwent Illumina RNA sequencing at 48 h post-irradiation to identify differentially expressed genes (DEGs) vs. controls. Systemic bias between groups was minimal, confirming stable global expression profiles (Figure 3a). Statistical thresholds (fold change >2, *p* < 0.05) identified 447 upregulated and 891 downregulated genes (total 1,338 DEGs). Functional and pathway enrichment analyses revealed associations with diverse biological processes, cellular components, and molecular functions (Figure 3b). Z-score-normalized clustering demonstrated distinct metabolic profiles between irradiated and control groups (Figure 3c). GO analysis highlighted significant enrichment in nucleocytoplasmic division and DNA double-strand break repair pathways (Figure 3d).

### 3.4 C-ion irradiation induces metabolomic changes in normal cells

**Figure 4:**
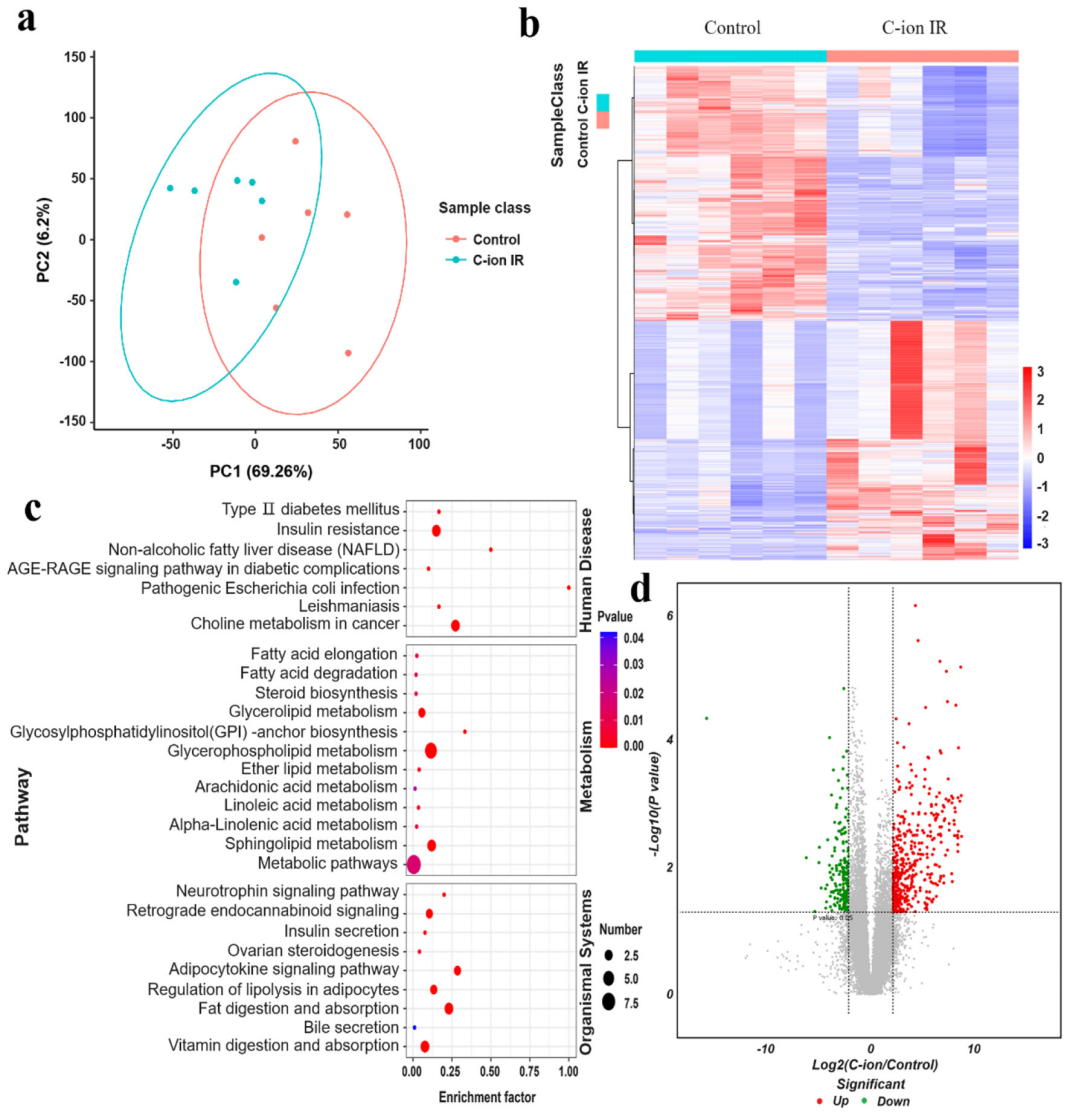
Metabolomic alterations analysis in the supernatant of Beas-2b cells 48 h after C-ion irradiation (2Gy). **(a)** PCA analysis of metabolites (*n* = 6). **(b)** Clustering analysis of differential metabolites (*n* = 6). **(c)** KEGG pathway enrichment of differential metabolites. **(d)** Differential metabolite analysis post-irradiation (*n* = 6).

Beas-2B normal cells irradiated with 2 Gy carbon ions were analyzed for metabolomic alterations. High biological reproducibility and systematic metabolic disparities were observed between the irradiated and control groups (Figure 4a). Carbon ion exposure induced significant metabolic shifts (Figure 4b). Standardized computational thresholds identified 201 differential metabolites (DMs) as potential biomarkers distinguishing irradiated from control samples. Functional enrichment analysis of these 201 DMs highlighted prominent associations with central carbon metabolism in cancer, fructose and mannose metabolism, and the arachidonic acid metabolism pathway (Figure 4c). Comparative analysis revealed 114 upregulated and 87 downregulated metabolites following irradiation (Figure 4d).

### 3.5 Integrated analysis of metabolomics and transcriptomics data

To systematically investigate the effects of C-ion irradiation on LUAD, MetaboAnalyst 6.0 joint pathway analysis module was employed to integrate 201 key DMs and 1,338 DEGs through shared metabolic pathways, enabling comprehensive biological pathway analysis (Table 1). This analysis identified three prominent pathways enriched with significant gene-metabolite interactions: arachidonic acid metabolism, fatty acid β-oxidation, and cholesterol metabolism.

**Table 1:** Differentially expressed genes (DEGs) associated with metabolic pathways identified through joint pathway analysis.

Gene	FDR	Log2 FC	Gene description	Enriched pathway
CPT1	3.14E-04	3.14	Carnitine Palmitoyltransferase 1	Arachidonic acid metabolism, Fatty acid β-oxidation, Sphingolipid metabolism pathway
EPAS1	2.15E-05	2.18	Endothelial PAS Domain Protein 1; Hypoxia-Inducible Factor 2 Alpha	Triglyceride metabolism pathway, Cholesterol metabolism pathway
GCH1	0.0031	−1.67	GTP cyclohydrolase 1	Fatty acid metabolism pathway, Phospholipid metabolism pathway, Cholesterol metabolism pathway

### 3.6 Validation of three core gene expression databases

**Figure 5:**
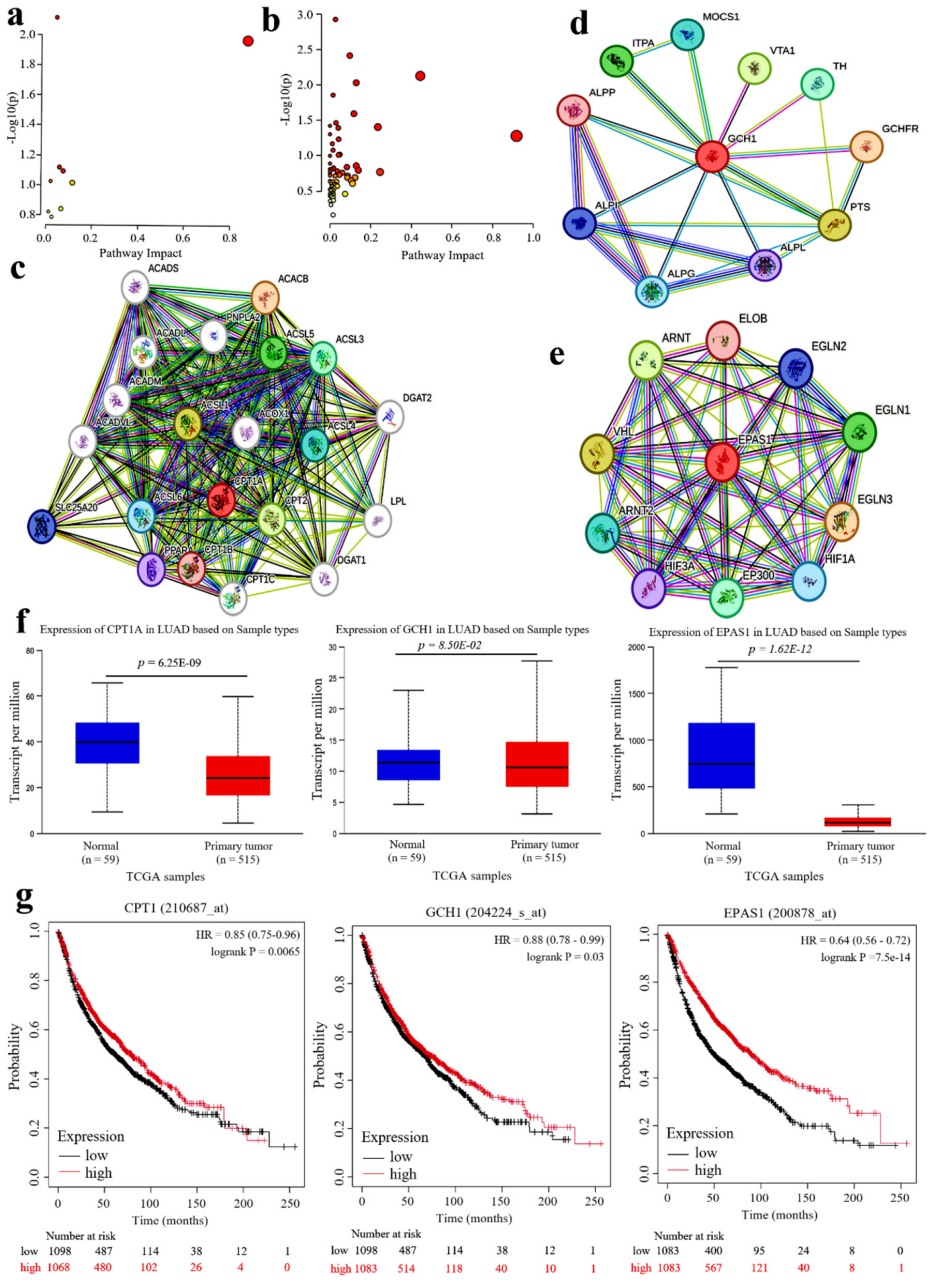
Integrated multi-omics screening and public database analysis of critical gene expression. **(a**, **b)** Metabolic pathway enrichment plot. **(c–e)** PPI network analysis of CPT1, GCH1 and EPAS1 genes. **(f)** Significantly expressed six genes in LUAD samples compared to normal samples. **(g)** Overall survival (OS) data evaluating the prognostic value of CPT1, GCH1 and EPAS1 in LUAD patients using Kaplan-Meier plotter. Bars represent mean ± SD.

Integrated analysis of metabolomic and transcriptomic datasets (Figures 5a, b) identified CPT1, GCH1, and EPAS1 as key regulators in C-ion radiation-mediated suppression of LUAD proliferation. To further explores the functional implications, a protein-protein interaction (PPI) subnetwork was constructed using the STRING database. The analysis yielded the following functional annotations: CPT1 was primarily associated with unsaturated fatty acid metabolism (Figure 5c), GCH1 regulated ROS homeostasis by modulating folate metabolism (Figure 5d), and EPAS1 played a central role in systemic oxygen homeostasis (Figure 5e). Using the GEPIA platform, mRNA expression of these genes was assessed in 515 LUAD and 55 normal lung tissue samples. CPT1 and EPAS1 exhibited significant downregulation in LUAD vs. normal tissues (*p* < 0.01), while GCH1 showed no significant change (*p* > 0.05) (Figure 5f). Kaplan-Meier survival analysis via the Kaplan-Meier Plotter demonstrated that high expression of CPT1 (HR = 0.85, 95% CI 0.75–0.96, log-rank *p* = 0.0065), GCH1 (HR = 0.88, 95% CI 0.78–0.99, *p* = 0.03), and EPAS1 (HR = 0.64, 95% CI 0.56–0.72, *p* = 7.5e-14) correlated with improved overall survival (OS) in LUAD patients (Figure 5g). These findings nominate CPT1, GCH1, and EPAS1 as potential prognostic biomarkers for C-ion radiotherapy in LUAD.

### 3.7 Validation of C-ion radiation on core gene expression

**Figure 6:**
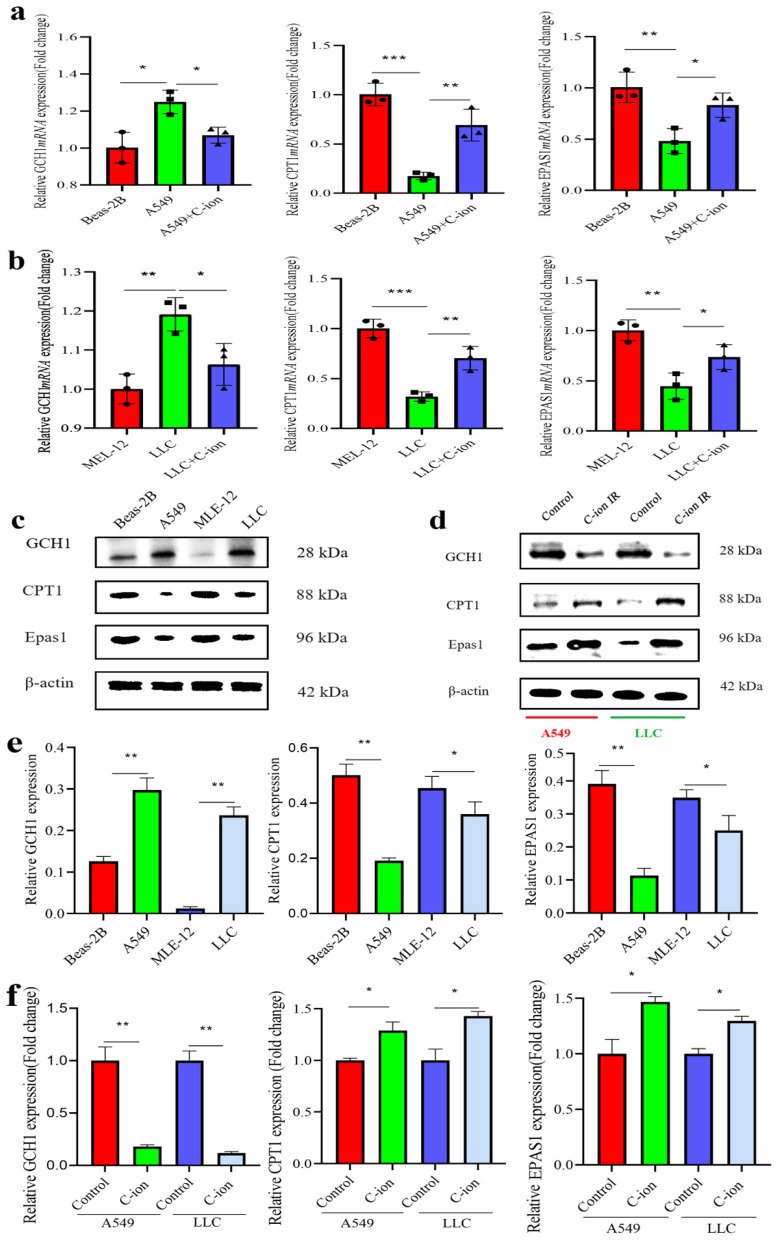
Validation of C-ion radiation effects on CPT1, GCH1, and EPAS1 expression across cell lines. **(a)** mRNA levels in Beas-2B cells, A549 cells, and A549 cells at 48 h after C-ion irradiation (5.16 Gy) (*n* = 3). **(b)** mRNA levels in MLE-12, LLC cells, and LLC cells at 48 h after C-ion irradiation (5.16 Gy) (*n* = 3). **(c)** Protein expression in normal and lung adenocarcinoma cell lines (*n* = 3). **(d)** Protein expression in irradiated vs. non-irradiated lung adenocarcinoma cells (*n* = 3). **(e**, **f)** Quantitative analysis of CPT1, GCH1, and EAPS1 protein in A549 and LLC cells (*n* = 3). Bars represent mean ± SD. *p < 0.05, **p < 0.01, ***p < 0.001 vs. control.

We examined the expression of three core genes (*CPT1, GCH1*, and *EPAS1*) in four cell lines irradiated with 5.16 Gy of carbon ions. Under baseline conditions, *CPT1* and *EPAS1* mRNA levels were significantly lower, whereas *GCH1* mRNA expression was significantly higher, in A549 and LLC lung adenocarcinoma cells compared with normal lung epithelial cells (BEAS-2B and MLE-12) (*p* < 0.01; Figures 6a, b). Following carbon-ion irradiation, *CPT1* and *EPAS1*mRNA levels increased significantly, while *GCH1* mRNA expression decreased significantly in both adenocarcinoma cell lines (*p* < 0.05; Figures 6a, b). Protein analysis confirmed that, relative to normal cells, baseline GCH1 protein expression was elevated, whereas CPT1 and EPAS1 expression were reduced in A549 and LLC cells (Figure 6c). Quantitative protein analysis demonstrated that, compared with normal cells (BEAS-2B and MLE-12), GCH1 protein levels were significantly higher and CPT1 and EPAS1 levels were significantly lower in tumor cells (*p* < 0.05; Figures 6d, e). Compared with the unirradiated control, C-ion irradiation significantly decreased GCH1 protein expression and significantly increased CPT1 and EPAS1 expression in tumor cells (A549 and LLC) (*p* < 0.05; Figure 6f).

### 3.8 Validation of C-ion radiation of core gene expression in tumors *in vivo*

**Figure 7:**
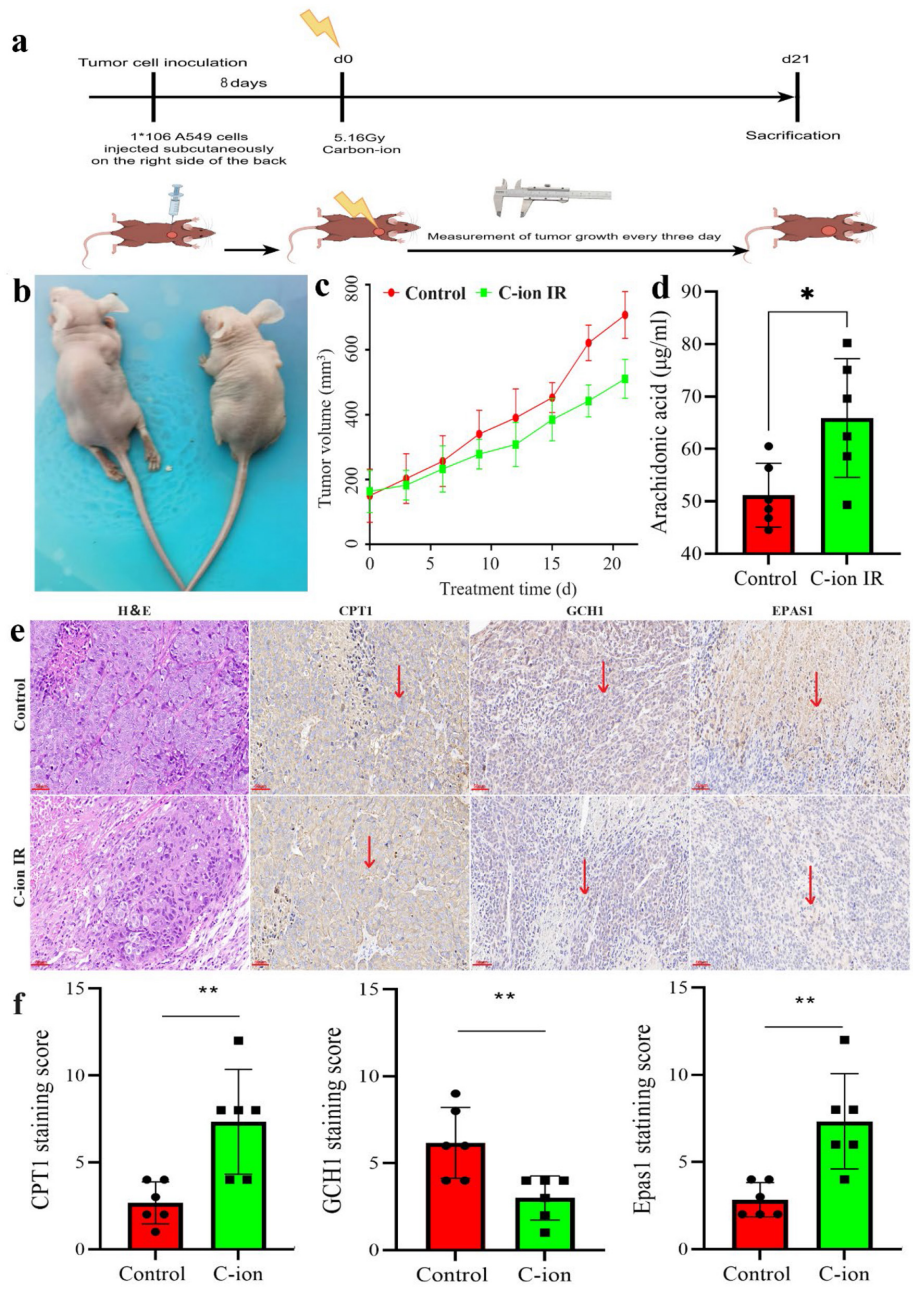
*In vivo* validation of CPT1, GCH1, and EPAS1 expression in tumor tissue 21 days after C-ion irradiation (5.16 Gy) (×400 magnification). **(a)** The timeline of inoculation, irradiation, measurement of tumor volumes, and analysis for radiotherapy. **(b)** Subcutaneous tumor model established on the right flank of nude mice. **(c)** Longitudinal monitoring of tumor volume changes. **(d)** Arachidonic acid (AA) levels in peripheral blood post-irradiation. **(e)** Representative H&E staining and immunohistochemical images of CPT1, GCH1, and EPAS1 in tumor sections. **(f)** Quantitative expression analysis of CPT1, GCH1, and EPAS1 in tumor tissues. *p < 0.05, **p < 0.01 vs. Control. Bars represent mean ± SD.

Tumor-bearing mouse model establishment finished in 8 days (Figure 7a). A549 xenograft models were established in BALB/c nude mice and subjected to 5.16 Gy C-ion irradiation (Figure 7b). From 9 days after irradiation, compared with the control group, C-ion groups could significantly inhibit tumor growth (Figure 7b), the tumor volumes at 21 days after treatment in each group were: 707.20 ± 72.15 mm3 in the control group, 510.21 ± 60.23 mm^3^ in the C-ion irradiation group. The detection of AA content in the serum of mice revealed that the AA content changed significantly after CIR, and the AA content in the body of mice increased significantly after 5.16 Gy CIR (Figures 7c, d). CPT1, GCH1, and EPAS1 immunostaining were detected in carcinoma cells in the tumor tissues (Figure 7e). Compared with the control group, carbon-ion irradiation significantly reduced GCH1 expression and promoted CPT1 and EPAS1 expression (Figure 7f).

## 4 Discussion

Radioresistance to conventional photon radiotherapy remains a critical barrier to improving survival in NSCLC (17). C-ion radiotherapy, an emerging modality leveraging unique physical and biological advantages, has demonstrated superior efficacy in treating diverse solid tumors. While our previous studies established C-ion irradiation as a potent inhibitor of tumor proliferation (18, 19), its precise molecular mechanisms in LUAD particularly multi-omics-driven regulatory networks are not fully elucidated. Carbon ion radiation induces DNA double-strand breaks and inhibits cell proliferation. This study systematically deciphers the anti-tumor mechanisms of C-ion irradiation in lung adenocarcinoma through integrated transcriptomic, proteomic, and metabolomic analyses. Studies have shown that C-ions exert direct cytotoxic effects via DNA double-strand break induction and cell cycle arrest (20, 21), while concurrently modulating oxidative stress and energy metabolism pathways (22). These findings provide novel molecular insights into CIRT applications for LUAD and establish a theoretical framework for refining radiotherapeutic strategies. This study demonstrates that C-ion irradiation not only significantly inhibits the proliferation of lung adenocarcinoma cells but also induces lipid metabolic alterations in normal lung cells, both of which collectively suppress tumor growth. C-ion radiation inhibits the migration and invasion of lung adenocarcinoma cells. Cell migration is predominantly dependent on cytoskeletal dynamics and adhesion cycles. In contrast, cell invasion necessitates the extra capability of extracellular matrix degradation. LLC cells were found to possess prominent migratory potential in the present study, a phenomenon potentially linked to the expression levels of matrix metalloproteinases and chemokine receptors (23). Interestingly, combined C-ion irradiation of lung adenocarcinoma and normal cells markedly enhances anti-proliferative effects. Screening identified multiple radiation-responsive genes, including CPT1, GCH1, and EPAS1, which may play important roles in radiation sensitivity. Existing evidence highlights glutathione peroxidase 4 and GCH1 as critical regulators of lipid peroxide scavenging (24). GCH1 prevents ferroptosis via its metabolites tetrahydrobiopterin (BH4) and dihydrobiopterin (25). T-cell-specific GCH1 knockout impairs proliferation and mitochondrial respiration, while GCH1 overexpression enhances BH4 synthesis, promoting tumor-infiltrating CD4^+^/CD8^+^ T-cell expansion and anti-tumor activity (26). C-ion irradiation reduced GCH1 expression, induced DNA damage, and promoted apoptosis in lung adenocarcinoma cells. BH4 exerts radioprotective effects by scavenging free radicals, facilitating the DNA damage response, and mitigating inflammatory responses (27). Consistent with this protective role, pharmacological inhibition of GCH1, which markedly lowers BH4 levels, significantly exacerbated ionizing radiation-induced endothelial nitric oxide synthase uncoupling and apoptosis (28). Radiation injury models and lung-specific Gch1 knockout/knock in mice reveal BH4 metabolism's pivotal role in radiation-induced ROS generation and radiosensitivity. Studies have shown that GCH1 overexpression elevates BH4 levels, amplifying CD4^+^/CD8^+^ T-cell responses and *in vivo* anti-tumor efficacy (29). GCH1 is identified as a ferroptosis suppressor-its knockout in KRAS-driven lung cancer models reduces tumor burden and prolongs survival (30). Aligning with our observations, post-irradiation GCH1 downregulation in lung adenocarcinoma confirms its pro-tumorigenic role in disease progression. This study found that the expression level of GCH1 in lung adenocarcinoma decreased after C-ion irradiation, which may inhibit the proliferation of lung adenocarcinoma cells. However, database analysis revealed that high GCH1 expression was associated with long-term survival benefit in patients, which may be primarily attributed to the high expression of GCH1 in the tumor immune microenvironment, leading to the effect of enhanced immune surveillance outweighing the proliferative signals of the cancer cells themselves. The transient downregulation induced by radiotherapy contrasts with the global immune status reflected by baseline high expression.

C-ion irradiation upregulates EPAS1 expression, with high EPAS1 levels correlating with improved prognosis in NSCLC patients. EPAS1 maintains the DNA repair capacity of hypoxic tumor cells primarily by transcriptionally upregulating key DNA damage response genes, including RAD51 and BRCA1, which in turn promote homologous recombination (HR) repair through multiple molecular interactions. Consequently, EPAS1 knockdown impairs HR and sensitizes hypoxic tumor cells to ionizing radiation (31). EPAS1 emerges as a critical therapeutic target—its overexpression enhances anti-tumor capacity in aged TCR-T cells (32). The EPAS1-targeted inhibitor YQ-0629 synergistically inhibits tumor growth with paclitaxel *in vitro* and *in vivo* (33), consistent with our findings. Notably, EPAS1 depletion attenuates GPX4 inhibitor-induced ferroptosis. Mechanistically, HIF-2α increases ferroptosis susceptibility in clear cell carcinoma by activating hypoxia-driven lipid droplet-associated proteins and suppressing GPX4 expression (34), potentially explaining context-dependent EPAS1 functionality across tumor types.

This study found that CPT1 expression is increased in NSCLC cells compared to normal lung epithelial cells, with C-ion irradiation further reducing CPT1 in lung adenocarcinoma. Existing studies confirm CPT1A drives ferroptosis resistance and CD8^+^ T-cell dysfunction in lung cancer (35). Studies on DNA damage repair have demonstrated that the percentage of γH2AX-positive nuclei is markedly decreased in *Cpt1b*-deficient models, and that inhibition of fatty acid oxidation can attenuate DNA damage or stimulate DNA repair. Moreover, in nasopharyngeal carcinoma, *CPT1A* expression levels are positively correlated with DNA replication and pyrimidine metabolism pathways (36). CPT1A knockdown significantly attenuates tumor growth and weight *in vivo* (36), establishing its role as an oncogenic driver in cancer progression and metastasis (35). Clinically, high CPT1 correlates with improved NSCLC prognosis, while radioresistant nasopharyngeal carcinoma exhibits enhanced fatty acid oxidation and elevated CPT1A expression linked to reduced post-radiotherapy survival (37). *In vivo*, C-ion irradiation inhibited tumor growth of NSCLC, and in xenograft tumor tissues, the expression of GCH1 was decreased, while the expression of CPT1 and EPAS1 was increased. Lipidomic profiling identifies multiple tumor-modulating metabolites, including diethyl glutarate, which increases cytotoxic CD8^+^ T-cell infiltration in tumors and periphery (38). Our study indicates that carbon-ion irradiation elevates the levels of AA, 3-hydroxytetradecanoic acid, and 2-methylglutaric acid in tumor-bearing mice, which may be involved in the antitumor immune response. In CRC, targeting AA metabolism enriches tumor microenvironmental CD8^+^ T cells. LTA4H, critical for AA-derived leukotriene B4 production, correlates with poor ovarian cancer prognosis (39). Dietary supplementation with α-linolenic acid, AA, or eicosapentaenoic acid potentiates anti-PD-L1 therapy efficacy (40). We propose that C-ion irradiation not only significantly suppresses tumor growth but also elevates AA levels in the normal tissues surrounding the tumor; these two mechanisms may synergistically inhibit the proliferation of LUAD. In this study, carbon-ion radiation induced alterations in lipid metabolites-including -AA, arginine, and tryptophan-in normal lung epithelial cells. We hypothesize that these metabolic changes may contribute to radiation-induced bystander effects in lung adenocarcinoma, with particular relevance to the roles of AA and unsaturated fatty acids in tumor proliferation and metastasis. Notably, metastatic tumors exhibit higher levels of PUFAs and lower levels of monounsaturated fatty acids compared with primary tumors. Membrane PUFAs are recognized as key drivers of ferroptosis (41), and emerging evidence suggests that targeting AA metabolism suppresses VEGF signaling, thereby mediating tumor metastasis (42). Furthermore, radiation-induced interferon-gamma (IFNγ) has been shown to synergize with AA to promote ferroptosis in tumor cells, inhibiting tumor progression (43).

In summary, through multi-omics analysis, this study confirmed both the direct and indirect effects of C-ion radiation in suppressing tumorigenesis, and preliminarily identified potential diagnostic biomarkers and therapeutic targets associated with the biological effects of high LET radiation in NSCLC. Nevertheless, several limitations should be acknowledged. First, the detailed mechanisms of the candidate biomarkers have yet to be validated in additional cell lines. Second, the interplay between C-ion radiation and the immune response in NSCLC remains to be explored, particularly in immunocompetent mouse models or clinical specimens. These unresolved issues will inform the direction of our future research, in which we aim to conduct multidimensional validation-across diverse cell lines, organoids, and tumor models-to further elucidate the underlying mechanisms.

## Data Availability

The original contributions presented in the study are included in the article/Supplementary material, further inquiries can be directed to the corresponding authors.
